# In vivo retention of ^18^F-AV-1451 in corticobasal syndrome

**DOI:** 10.1212/WNL.0000000000004264

**Published:** 2017-08-22

**Authors:** Ruben Smith, Michael Schöll, Håkan Widner, Danielle van Westen, Per Svenningsson, Douglas Hägerström, Tomas Ohlsson, Jonas Jögi, Christer Nilsson, Oskar Hansson

**Affiliations:** From the Departments of Neurology (R.S., H.W., C.N.), Clinical Neurophysiology (D.H.), Radiation Physics (T.O.), and Clinical Physiology and Nuclear Medicine (J.J.), Skåne University Hospital (D.v.W.), Lund; Clinical Memory Research Unit (R.S., M.S., C.N., O.H.), Department of Clinical Sciences (D.v.W.), and Department of Diagnostic Radiology (D.v.W.), Lund University, Malmö; Wallenberg Centre for Molecular and Translational Medicine and the Department of Psychiatry and Neurochemistry (M.S.), University of Gothenburg; Department of Clinical Neuroscience (P.S.), CMM L8:01, Stockholm; and Memory Clinic (O.H.), Skåne University Hospital, Malmö, Sweden.

## Abstract

**Objective:**

To study the usefulness of ^18^F-AV-1451 PET in patients with corticobasal syndrome (CBS).

**Methods:**

We recruited 8 patients with CBS, 17 controls, 31 patients with Alzheimer disease (AD), and 11 patients with progressive supranuclear palsy (PSP) from the Swedish BioFINDER study. All patients underwent clinical assessment, ^18^F-AV-1451 PET, MRI, and quantification of β-amyloid pathology. A subset of participants also underwent ^18^F-FDG-PET.

**Results:**

In the 8 patients with CBS, 6 had imaging findings compatible with the corticobasal degeneration pathology and 2 with typical AD pathology. In the 6 patients with CBS without typical AD pathology, there were substantial retentions of ^18^F-AV-1451 in the motor cortex, corticospinal tract, and basal ganglia contralateral to the most affected body side. These patients could be clearly distinguished from patients with AD dementia or PSP using ^18^F-AV-1451. However, cortical atrophy was more widespread than the cortical retention of ^18^F-AV1451 in these CBS cases, and cortical AV-1451 uptake did not correlate with cortical thickness or glucose hypometabolism. These results are in sharp contrast to AD dementia, where ^18^F-AV-1451 retention was more widespread than cortical atrophy, and correlated well with cortical thickness and hypometabolism.

**Conclusions:**

Patients with CBS without typical AD pathology exhibited AV-1451 retention in the motor cortex, corticospinal tract, and basal ganglia contralateral to the affected body side, clearly different from controls and patients with AD dementia or PSP. However, cortical atrophy measured with MRI and decreased ^18^F-fluorodeoxyglucose uptake were more widespread than ^18^F-AV-1451 uptake and probably represent earlier, yet less specific, markers of CBS.

**Classification of evidence:**

This study provides Class III evidence that ^18^F-AV-1451 PET distinguishes between CBS and AD or PSP.

Corticobasal degeneration (CBD) pathology gives rise to a variety of clinical presentations that encompass (1) the classic presentation, known as corticobasal syndrome (CBS); (2) a syndrome similar to progressive supranuclear palsy (CBD-PSP); (3) a frontotemporal behavioral variant; and (4) a variant with progressive nonfluent aphasia (CBD-PNFA).^1^ However, clinical symptoms similar to the ones described above can also be caused by PSP, Alzheimer disease (AD), and non-tau pathologies, making an accurate prediction of the underlying pathology difficult in vivo.^2,3^

Tau PET tracers were developed to detect AD tau pathology, i.e., paired helical filaments with a mixture of 3R and 4R tau isoforms.^4^ In CBD, the main isoform is 4R-tau, with a straight filament ultrastructure, and the ability of ^18^F-AV-1451 to bind to these inclusions has been debated.^5–7^ Using autoradiography in postmortem tissue, some, but not all, studies show a low, yet specific, binding of AV-1451 in patients with CBD.^5–7^ Uptake of ^18^F-AV-1451 in vivo correlated with postmortem levels of tau pathology in 2 recently published autopsy cases of CBD,^8,9^ but the standardized uptake value ratios (SUVRs) were generally lower than in AD.

The aim of the present study was to investigate the pattern of in vivo retention of ^18^F-AV-1451 in patients with CBS, and how this pattern differs from the ones observed in patients with PSP and AD. We investigated 8 patients with CBS, 17 controls, 11 patients with PSP, and 31 patients with AD. The participants underwent ^18^F-AV1451 PET, ^18^F-FDG PET, structural MRI, and CSF or ^18^F-flutemetamol assessment of β-amyloid (Aβ) pathology.

## METHODS

### Primary research question.

Is the regional uptake of ^18^F-AV-1451 PET different in patients with CBS when compared to patients with PSP or AD? This study provides Class III evidence that ^18^F-AV-1451 PET distinguishes between CBS and AD or PSP.

### Participants.

The study participants were recruited from neurology and memory clinics in the southern part of Sweden as part of the Swedish BioFINDER study (www.biofinder.se). In the present study, we included 17 neurologically healthy, age-matched controls, 8 patients with CBS, 11 patients with PSP, and 31 patients with AD. The patients with CBS fulfilled the modified Cambridge criteria of CBS.^10^ Further, they also fulfilled the criteria for possible or probable CBD according to the Armstrong criteria,^1^ where 7 of the included patients were diagnosed with the CBS phenotype and 1 patient was diagnosed with possible CBD of the PNFA subtype.^1^ Nine of the patients with PSP were diagnosed with Richardson syndrome according to the National Institute of Neurological Disorders and Stroke criteria^11^ and 2 patients were diagnosed with PSP parkinsonism.^12^ Patients with AD dementia met DSM-III-R criteria for dementia as well as National Institute of Neurological and Communicative Disorders and Stroke–Alzheimer’s Disease and Related Disorders Association criteria for AD.^13^ All patients with AD dementia had pathologic amyloid status as confirmed by lumbar puncture or ^18^F-flutemetamol PET as previously described.^14^ All participants with AD dementia also fulfilled the National Institute on Aging–Alzheimer’s Association criteria for probable AD dementia with evidence of the AD pathophysiologic process.^15^

Since evidence of Aβ pathology has been suggested as an exclusion criterion for CBS diagnosis, amyloid status was determined using CSF measures of Aβ_42_ or ^18^F-flutemetamol PET as previously described.^14,16^ The patients were assessed with Hoehn & Yahr staging,^17^ Schwab and England activities of daily living, Mini-Mental State Examination, and detailed neurologic examination by a physician experienced in movement disorders.

### Standard protocol approvals, registrations, and patient consents.

Informed written consent was obtained from all patients before inclusion in the study. All procedures conformed to the Declaration of Helsinki and were approved by the regional ethics committee at Lund University, the Radiation Protection Committee at Skåne University Hospital, and the Swedish Medical Products Agency.

### MRI.

All patients underwent MRI on a 3.0T Siemens Skyra scanner (Siemens Medical Solutions, Erlangen, Germany), acquiring a T1-weighted magnetization-prepared rapid gradient echo. The cortical thickness in each region in figures e-1 and e-2 (at Neurology.org) was determined using FreeSurfer 5.3 segmentation^18^ of the MRI and using the Desikan-Killiany atlas.^19^ Methods for voxelwise analysis of MRI data are provided in the e-Methods.

### PET acquisition and data analysis.

The radiosynthesis procedure, radiochemical purity, and scanning methods for ^18^F-AV-1451 have been described in detail previously.^20^ The majority of patients and controls underwent the simplified protocol including only one dynamic ^18^F-AV-1451 scan for 80–120 minutes (8 × 300 seconds frames) postinjection. PET data were further processed using an in-house developed pipeline described earlier,^21^ based on FreeSurfer 5.3 segmentation of the MRI, and SUVR calculation using cerebellar gray matter as the reference region for ^18^F-AV-1451 and a composite pons/brainstem reference region for ^18^F-fluorodeoxyglucose (FDG). FDG and flutemetamol PET acquisitions and analyses were performed as previously described.^21^

**Figure 1:**
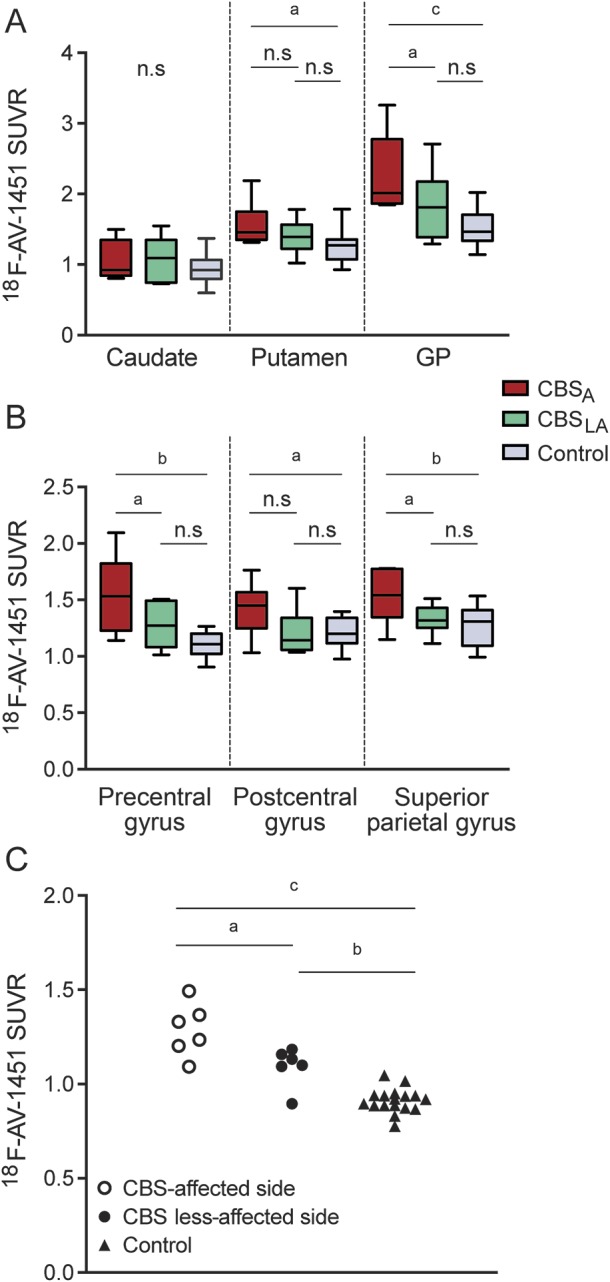
^18^F-AV-1451 retention in different brain regions Standardized uptake value ratios (SUVRs) in the basal ganglia (A) and the cerebral cortex (B). Results are shown for corticobasal syndrome (CBS), most affected side (CBS_A_); CBS, less affected side (CBS_LA_); and controls. (C) SUVRs in the most atrophic cortical region. GP = globus pallidus; n.s = not significant. ^a^*p* < 0.05, ^b^*p* < 0.01, ^c^*p* < 0.001.

Since the disease is asymmetric when it comes to clinical presentation and tau pathology, and since the most affected side varies from patient to patient, the data from patients with CBD have been compared between the affected and less affected side rather than between left and right sides where indicated. Control regions of interest (ROIs) in figure 1 were created by pooling the bilateral ROIs.

Methods for partial volume effects correction, analysis of SUVRs in atrophic cortex (figure 1C), analysis of the corticospinal tract, and voxelwise analysis of PET data are provided in the e-Methods.

### Statistics.

For comparisons between groups, Mann-Whitney *U* test was used. When comparing within the patients with CBD (affected/less affected side), Wilcoxon signed rank tests were used. For correlations, Pearson or Spearman correlations were performed where appropriate. Statistical significance was assumed at *p* < 0.05.

## RESULTS

### Participants.

The clinical data of the 8 patients with CBS are presented in table 1. The clinical data of controls, patients with AD dementia, and patients with PSP are presented in table e-1.

**Table 1:**
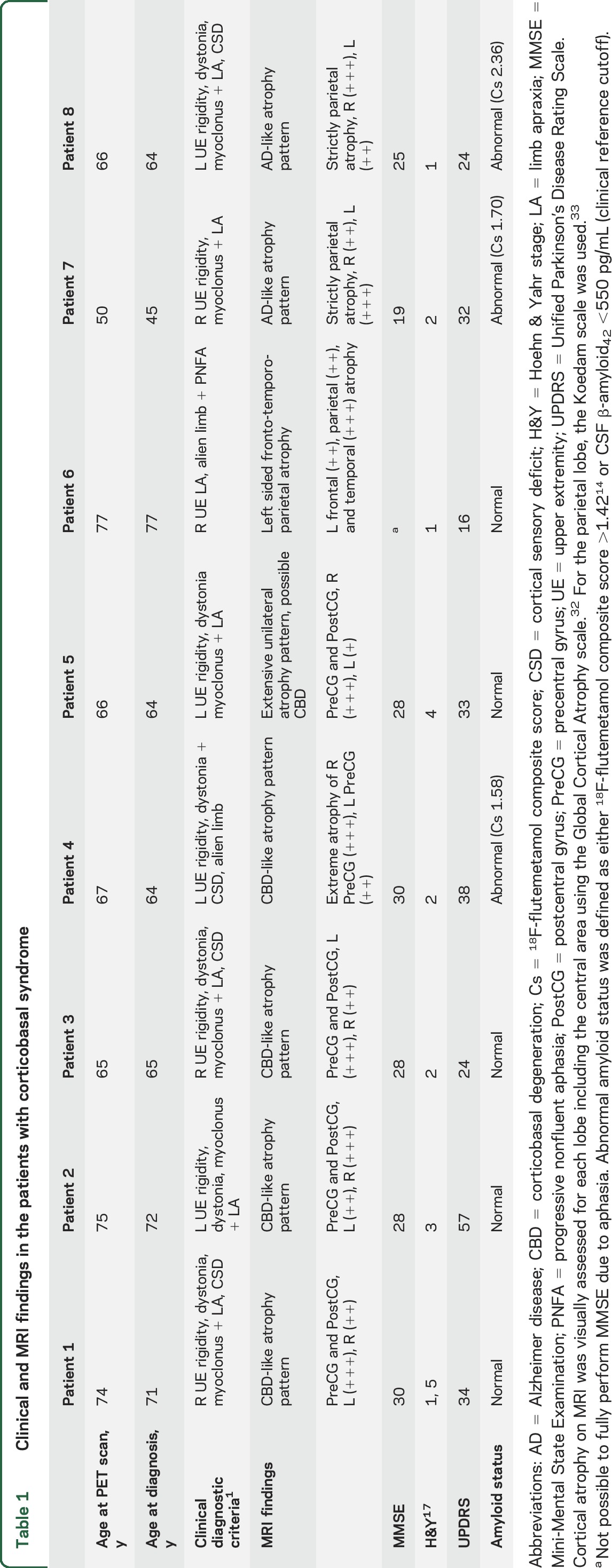
Clinical and MRI findings in the patients with corticobasal syndrome

### Distribution of ^18^F-AV-1451 retention in CBS and AD dementia.

**Figure 2:**
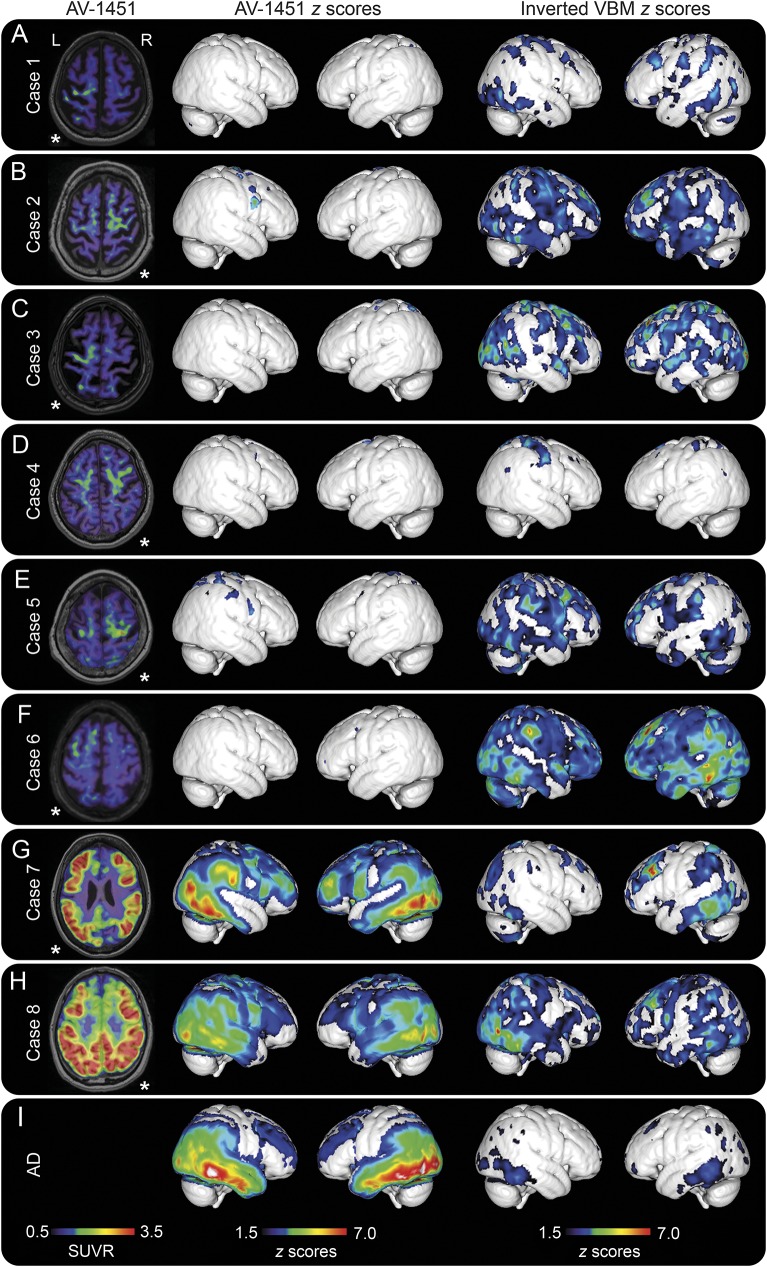
^18^F-AV-1451 PET, AV-1451 cortical *z* scores, and MRI cortical atrophy *z* scores (A–H) Cases 1 to 8, where A–E represent patients with corticobasal syndrome (CBS), F represents the patient with CBS with a progressive nonfluent aphasia phenotype, and G and H represent CBS cases with Alzheimer disease (AD) pathology. (I) Mean *z* score maps of the AD dementia control group (n = 31). The transversal images shown in the first column are in neurologic orientation, where left in the image corresponds to the patient’s left side. *The most affected side of the brain in each patient. *z* Scores are presented for values >1.5. SUVR = standardized uptake value ratio; VBM = voxel-based morphometry.

The uptake patterns of ^18^F-AV-1451 in our patients were diverse, which is expected considering the heterogeneity of pathologies underlying clinically diagnosed CBS (figure 2). Two patients had visually a bilateral and symmetric temporo-parietal atrophy with a relative sparing of the precentral gyri. In these 2 patients, the clinical presentation was dominated by cortical parietal symptoms (apraxia and visuospatial deficiencies) and asymmetric motor symptoms were present, but milder. These patients had a very prominent ^18^F-AV-1451 uptake in the temporo-parietal lobes, exhibiting a pattern very similar to that seen in patients with AD dementia (figure 2, G–I). In these cases, ^18^F-flutemetamol scans were clearly positive, indicative of an underlying AD pathology (table 1, cases 7 and 8). Since these patients had a different underlying pathology, where both ^18^F-flutemetamol and ^18^F-AV-1451 uptake were compatible with AD, they were excluded from subsequent group analyses.

**Figure 3:**
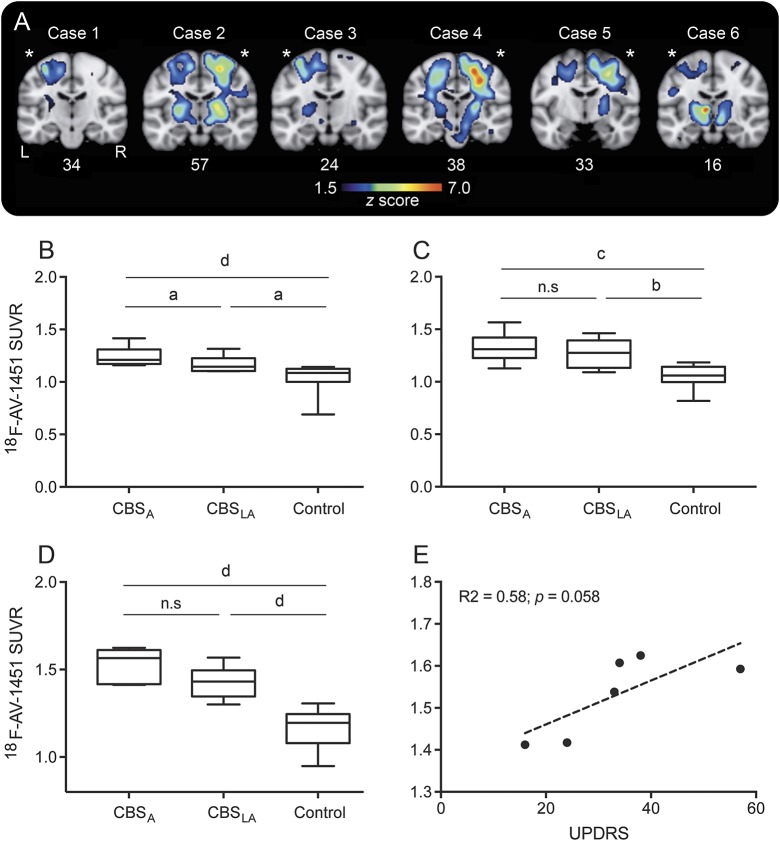
AV-1451 retention in the corticospinal tract (A) *z* Score maps of AV-1451 uptake in coronal slices transecting the brain. Numbers below the images indicate the Unified Parkinson’s Disease Rating Scale (UPDRS) part III (motor) score. *The most affected side of the brain in each patient. *z* Scores are presented for values 1.5–7. (B) Standardized uptake value ratios (SUVRs) in the corticospinal tract using the JHU white matter tract atlas. (C) SUVRs in the subcortical white matter underlying the precentral gyrus. (D) SUVRs in the posterior limb of the internal capsule, containing the corticospinal tract. (E) Spearman correlation between SUVRs in the internal capsule and the UPDRS part III. CBS_A_ = corticobasal syndrome most affected side; CBS_LA_ = corticobasal syndrome less affected side; n.s = not significant. ^a^*p* < 0.05, ^b^*p* < 0.01, ^c^*p* < 0.001, ^d^*p* < 0.0001.

The patients with clinical CBS with a non-AD pattern (n = 6) had visually an asymmetric cortical retention of ^18^F-AV-1451 in the motor areas contralateral to the most affected limb (figure 1B; figure 2, A–F; and figure 3). This retention corresponded to a cortical atrophy on MRI surrounding the central sulcus with the precentral gyrus being heavily atrophied (figure 1, A–E). The patient with CBS with a nonfluent aphasia (CBD-PNFA; case 6) also had a pronounced left fronto-temporo-parietal atrophy matching the clinical symptoms (figure 2F).

When comparing the cortical atrophy detected using MRI to the extent of AV-1451 uptake, we found that the cortical atrophy seen in the 6 patients with CBS with no typical AD pathology (inverted voxel-based morphometry *z* scores; figure 2, A–F) was more pronounced and widespread compared to the cortical retention of ^18^F-AV-1451 (AV-1451 *z* scores; figure 2, A–F). This was in contrast to the group with typical amnestic dementia due to AD pathology, where the *z*-score maps showed that ^18^F-AV-1451 retention was more widespread than cortical atrophy (figure 2I), indicating that ^18^F-AV-1451 is an earlier marker of pathology than MRI-based atrophy measures in AD dementia, but the opposite seems to be the case in CBS without typical AD pathology.

Next, we studied the uptake of ^18^F-AV-1451 in subcortical regions in the 6 CBS cases with no typical AD pathology and found the highest ^18^F-AV-1451 retention in the white matter underlying the atrophic cortical gyri as well as in the basal ganglia of the affected side of the brain (figure 3A). Interestingly, the subcortical tau pathology showed a distribution corresponding to the corticospinal tract (CST) in the affected hemisphere. Using a white matter tract atlas for the CST, we found a significant increase in the tract contralateral to the most affected side of the body compared to the ipsilateral side and to controls (figure 3B). Further, the CST was manually subdivided into the white matter underlying the precentral gyrus and the internal capsule to rule out that the results were merely an effect of the proximity of the CST to the basal ganglia. Similar findings were seen using this subdivision (figure 3, C and D). The intensity of retention in the internal capsule showed a trend for a correlation to Unified Parkinson’s Disease Rating Scale–III score severity (*p* = 0.058; figure 3E).

### Group differences in uptake of ^18^F-AV-1451 in patients with CBS vs controls.

**Figure 4:**
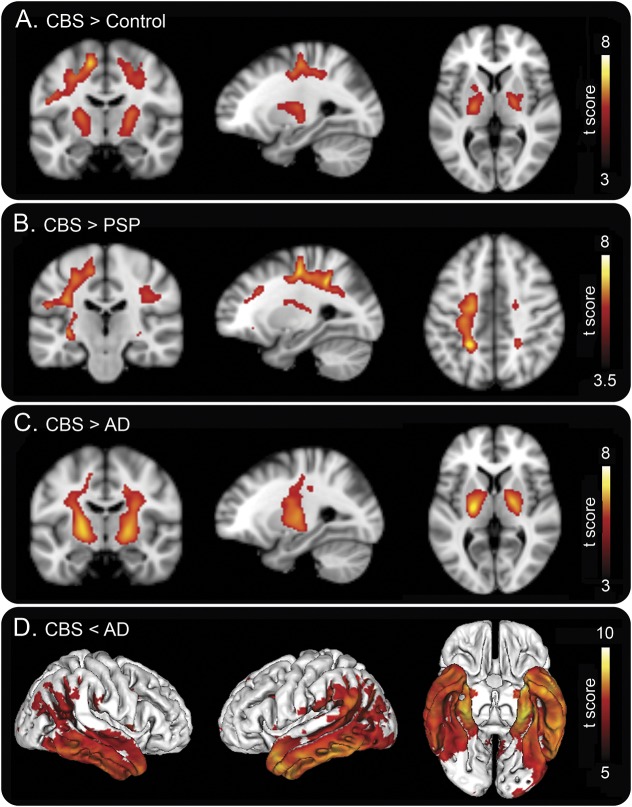
Voxel-based analysis of ^18^F-AV-1451 retention (A–C) Coronal, sagittal, and transversal sections show where the uptake is higher in corticobasal syndrome (CBS) compared to (A) controls, (B) patients with progressive supranuclear palsy (PSP), and (C) patients with Alzheimer disease (AD) (*p* < 0.001, uncorrected for multiple comparisons). (D) Lateral and inferior views of areas where the signal is higher in AD than in CBS (*p* < 0.05, corrected for family-wise error). Scales indicate *t* scores.

When comparing the retention of ^18^F-AV-1451 in frontal, temporal, and parietal regions, as well as the basal ganglia, in patients with CBS with no typical AD pathology vs healthy controls, we found clearly increased retention in the globus pallidus and putamen of the affected side (figure 1A; table e-2). Further, the SUVRs of AV-1451 were significantly increased in the precentral gyrus, postcentral gyrus, and superior parietal gyrus, but the group-level changes in these cortical regions were modest due to the heterogeneity in the regional uptake of AV-1451 in different cortical regions in these 6 CBS cases (figure 1B; table e-2). Whole brain voxel-based analyses revealed similar findings with increased retention of ^18^F-AV-1451 in the motor cortex, the CST, and the basal ganglia (figure 4A). However, to circumvent the problem with somewhat varying regions being the most affected by the pathology in different patients with CBS, we also delineated a ROI in the most atrophic cortical region, using the structural MRI, and a reference region on the contralateral side. Using this approach, we observed even greater differences in the cortical uptake of AV-1451 between the patients with CBS and controls (figure 1C; table e-2). Using an occipital lobe reference instead of a cerebellar reference for generating SUVR values resulted in similar findings (figure e-3).

### Group differences in uptake of ^18^F-AV-1451 in patients with CBS vs AD dementia and PSP.

Using a voxel-based approach, we compared the ^18^F-AV-1451 retention in our 6 patients with CBS vs patients with PSP or AD dementia. We found that the patients with CBS had increased uptake in the subcortical white matter underlying the motor cortex when compared to PSP and AD dementia, respectively (figure 4, B–C). Increased uptake was also present in the basal ganglia of patients with CBS when compared to patients with AD dementia (figure 4C). We also found that patients with AD dementia had clearly higher uptake of ^18^F-AV-1451 in temporoparietal cortical areas compared to patients with CBS (figure 4D). In the inferior temporal gyrus, the discrimination between AD dementia and CBS was highly significant (figure e-4).

### Comparison of AV-1451 with cortical atrophy and glucose metabolism.

Comparing the retention of ^18^F-AV-1451 to the MRI-based cortical thickness, we found weak inverse correlations between cortical thickness and ^18^F-AV-1451 retention in 3 out of the 6 patients with CBS, but strong inverse correlations in the 2 patients with CBS due to AD pathology (figure e-1).

^18^F-FDG PET scans were performed in 4 of our patients with CBS without typical AD pathology and the 2 patients with clinical CBS with biomarkers suggestive of AD pathology. In the patients with AD pathology, there were very strong correlations between increased retention of ^18^F-AV-1451 and reduced uptake of ^18^F-FDG, but a weak similar observation was only found in one of the participants with CBS without typical AD pathology (figure e-2). Further, in all CBS cases but one (case 4) we found weak but significant correlations between decreases in ^18^F-FDG uptake and increasing atrophy of the cerebral cortex (figure e-2).

## DISCUSSION

We found specific retention of ^18^F-AV-1451 in all cases with a clinical diagnosis of CBS. It is well-established that AD pathology can cause clinical symptoms equivalent to CBS^3^ and, indeed, 2 of the 8 clinical CBS cases had a bilateral temporoparietal uptake pattern similar to the pattern seen in AD, as well as positive ^18^F-flutemetamol scans. The other 6 cases with a clinical CBS phenotype exhibited ^18^F-AV-1451 retention in the motor cortex and subcortical white matter contralateral to the side of symptoms, where tau deposition is to be expected.^22–24^ Further, the patient with CBS with a nonfluent aphasia (CBD-PNFA; case 6) had a predominantly left sided frontotemporal uptake of the tracer, corresponding to the clinical symptoms. The ^18^F-AV-1451 SUVR values in the patients with CBS were lower than the values seen in the patients with AD dementia, but the retention was clearly higher than in controls. Our results are in line with data from 2 recent autopsy studies on 2 patients with CBD who were scanned using ^18^F-AV-1451 in life and where tau pathology was assessed neuropathologically postmortem.^8,9^ These studies suggest that there is an in vivo affinity of AV-1451 also for the 4R tau pathology in CBD, but this affinity is lower than for the 3R and 4R paired helical filaments of AD. This is in contrast to PSP, where the in vivo signal of AV-1451 does not seem to correlate with 4R tau pathology.^6,25^ Another recent study^22^ describes uptake of ^18^F-THK-5351 in the motor cortex, as well as subcortical white matter and basal ganglia contralateral to the affected side of the body in patients with CBS, very much in line with our results. Altogether, these studies suggest that it might be possible to assess the tau burden in CBD in vivo using ^18^F-AV-1451 or ^18^F-THK-5351.

A recently published MRI study has shown more microstructural changes of the white matter, including the CST, in patients with CBS with no typical AD pathology compared to both healthy controls and patients with CBS due to AD.^26^ We found AV-1451 retention in the cerebral cortex, but an even more pronounced retention was seen in the subcortical white matter as well as in the basal ganglia contralateral to the affected side of the body. This subcortical pathology is not visualized using cortical *z* scores, but is evident when looking at sections transecting the brain (figure 3).

The CBD pathology affects the frontal motor cortex, but also parietal cortex and subcortical structures such as the globus pallidus and substantia nigra.^24^ As discussed above, in the present study we show that the pattern of distribution of the cortical ^18^F-AV-1451 retention in suspected CBS mainly occurs in the motor cortex and subcortical gray and white matter contralateral to the side of symptoms. This is clearly different from the pattern observed in patients with clinically diagnosed PSP, where the uptake is most prominent bilaterally in the globus pallidus,^27–29^ and from the pattern in patients with AD dementia (figures 2I and 4), where the ^18^F-AV-1451 signal is most abundant in the temporal and parietal lobes.

Today, cortical atrophy on MRI and neuronal hypometabolism on FDG-PET are used in the diagnostic workup of CBS, but both markers of pathology appear relatively late in the disease process. We therefore studied whether ^18^F-AV-1451 PET changes could be an earlier marker of CBD pathology. The cortical AV-1451 retention in our 6 patients with CBS without typical AD pathology was present in areas with cortical atrophy, but the extent of the cortical atrophy on MRI exceeded the extent of the cortical AV-1451 retention. Moreover, ^18^F-FDG PET, but not ^18^F-AV-1451 PET, correlated with the amount of cortical atrophy found in these patients. Together, these results indicate that ^18^F-AV-1451 is a less sensitive measurement of cortical cell loss and dysfunction than MRI or ^18^F-FDG PET in CBS. Likely, the lower affinity of AV-1451 to the 4R pathology in CBD compared to the paired helical filaments in AD is in part responsible for this effect. In line with this, we found that the extent of the ^18^F-AV-1451 uptake was larger than the areas affected by cortical atrophy on MRI in our patients with AD dementia, indicating that MRI is less sensitive than ^18^F-AV-1451 in determining the regions affected by AD pathology in AD dementia. We further found strong inverse correlations between FDG uptake and AV-1451 retention in patients with AD dementia, in line with what we, and others, have previously published.^21,30,31^

Limitations of the present study include the sample size, with only 8 patients with CBS included. The low number of cases prevents us from making further subanalyses and limits the study to a case series. Further, the absence of neuropathologic confirmation of the CBD diagnoses limits the certainty of the conclusions. It is well-established that CBS can be caused by other underlying pathologies than CBD, such as AD or PSP.^3^ The present clinical diagnoses were based on clinical CBS criteria^1,10^ and we cannot rule out that pathologies other than CBD-related tau pathology may cause the clinical symptoms in the 6 non-AD cases with CBD-like symptoms. However, the pattern of uptake of ^18^F-AV-1451 in these 6 cases indicates that the underlying pathology might indeed be CBD, and at least not AD or typical PSP pathology. Another limitation is that some of the retention seen in the basal ganglia likely represents off-target binding.

Our results support the usefulness of ^18^F-AV-1451 PET as a marker of tau pathology in CBS, with cortical and subcortical retention seen in motor areas and the pyramidal tract contralateral to the affected side of the body and corresponding to clinical symptoms. Further, we found that cortical MRI changes and ^18^F-FDG-PET reductions are more widespread than the cortical retention of ^18^F-AV-1451. Therefore, MRI and FDG PET probably represent earlier, but less specific, markers of disease in CBS than AV-1451. Future studies are needed to determine whether accumulation of tau pathology over time in CBD can be reliably measured with ^18^F-AV-1451 PET and to determine its usefulness as an additional diagnostic tool and as an outcome measure in trials evaluating new disease-modifying therapies against CBD.

## Supplementary Material

Data Supplement

## GLOSSARY

Aβ: β-amyloid
AD: Alzheimer disease
CBD: corticobasal degeneration
CBS: corticobasal syndrome
CST: corticospinal tract
DSM-III-R: *Diagnostic and Statistical Manual of Mental Disorders, 3rd edition, revised*
FDG: ^18^F-fluorodeoxyglucose
PNFA: progressive nonfluent aphasia
PSP: progressive supranuclear palsy
ROI: region of interest
SUVR: standardized uptake value ratio
