# Supplementary material for: In vivo retention of 18F-AV-1451 in corticobasal syndrome
Source: Neurology. 2017 Aug 22;89(8):845–53. doi: 10.1212/WNL.0000000000004264 (PMC5580862; doi:10.1212/WNL.0000000000004264)
Supplement: Data Supplement [file supp_89_8_845__index.html]

In vivo retention of 18F-AV-1451 in corticobasal syndrome — Data Supplement 

# In vivo retention of 18F-AV-1451 in corticobasal syndrome

## Data Supplement

**Neurology® data supplements are not copyedited before publication. Published editorials and translations have been copyedited.  
 © 2017 American Academy of Neurology.  
  
 Files in this Data Supplement:**

- Figure e-1 - PDF
- Figure e-2 - PDF
- Figure e-3 - PDF
- Figure e-4 - PDF
- Table e-2 - PDF
- e-Methods - Microsoft Word file
- Table e-1 - Microsoft Word file
