# Supplementary material for: In vivo retention of 18F-AV-1451 in corticobasal syndrome
Source: Neurology. 2017 Aug 22;89(8):845–53. doi: 10.1212/WNL.0000000000004264 (PMC5580862; doi:10.1212/WNL.0000000000004264)
Supplement: Data Supplement [file supp_WNL.0000000000004264_Suppl_Figure_e-1.pdf]

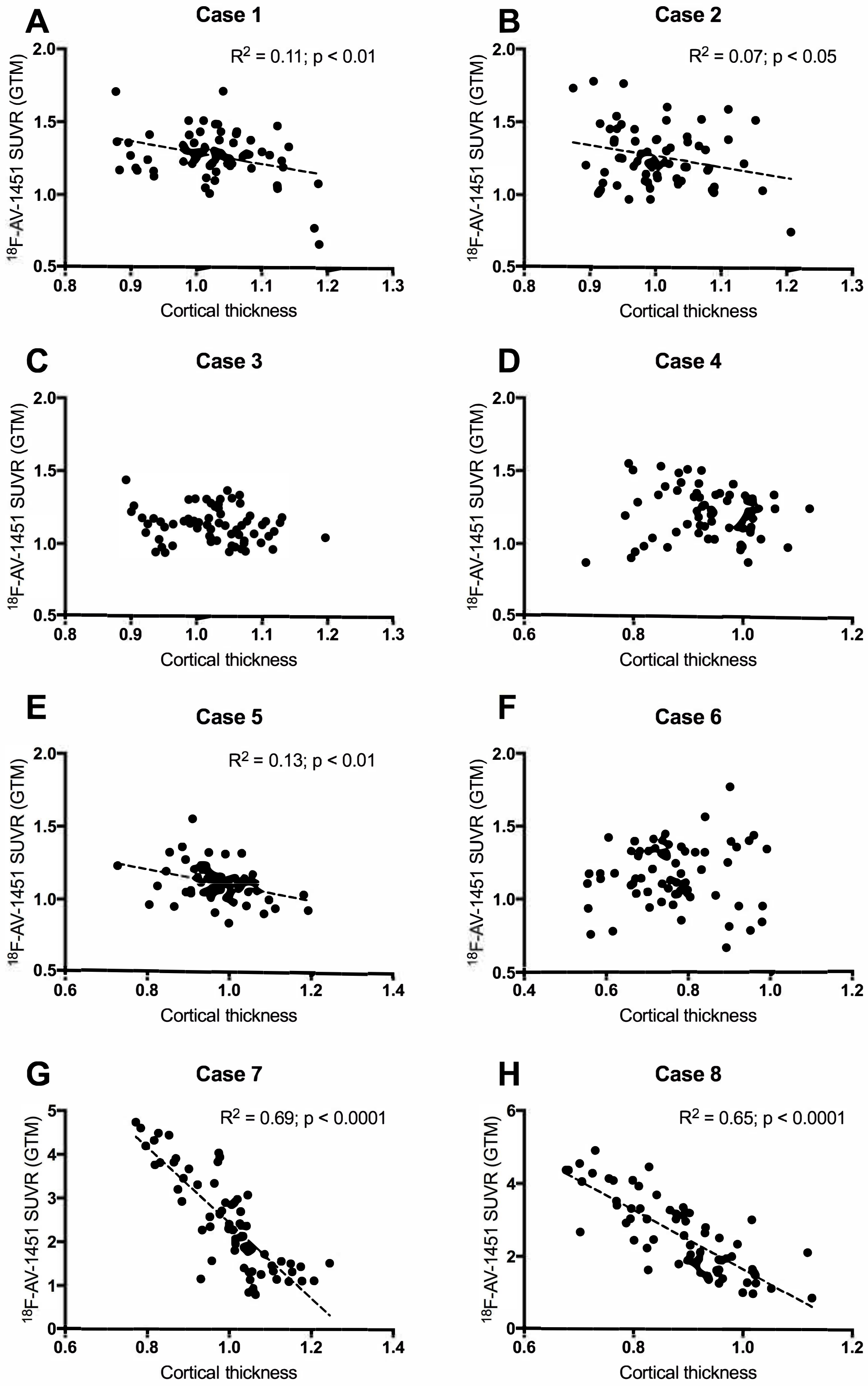


**Supplementary Figure e-1**.

Correlations between cortical thickness ratio (Patient cortical thickness/cortical thickness in controls) and $^{18}$F-AV-1451 PET SUVR. Inverse correlations were seen between cortical thickness and $^{18}$F-AV-1451 SUVRs in the AD-patients mimicking CBS (cases 7 and 8). Weak correlations were seen in CBS-cases 1, 2 and 5.
