# Supplementary material for: In vivo retention of 18F-AV-1451 in corticobasal syndrome
Source: Neurology. 2017 Aug 22;89(8):845–53. doi: 10.1212/WNL.0000000000004264 (PMC5580862; doi:10.1212/WNL.0000000000004264)
Supplement: Data Supplement [file supp_WNL.0000000000004264_Suppl_Figure_e-2.pdf]

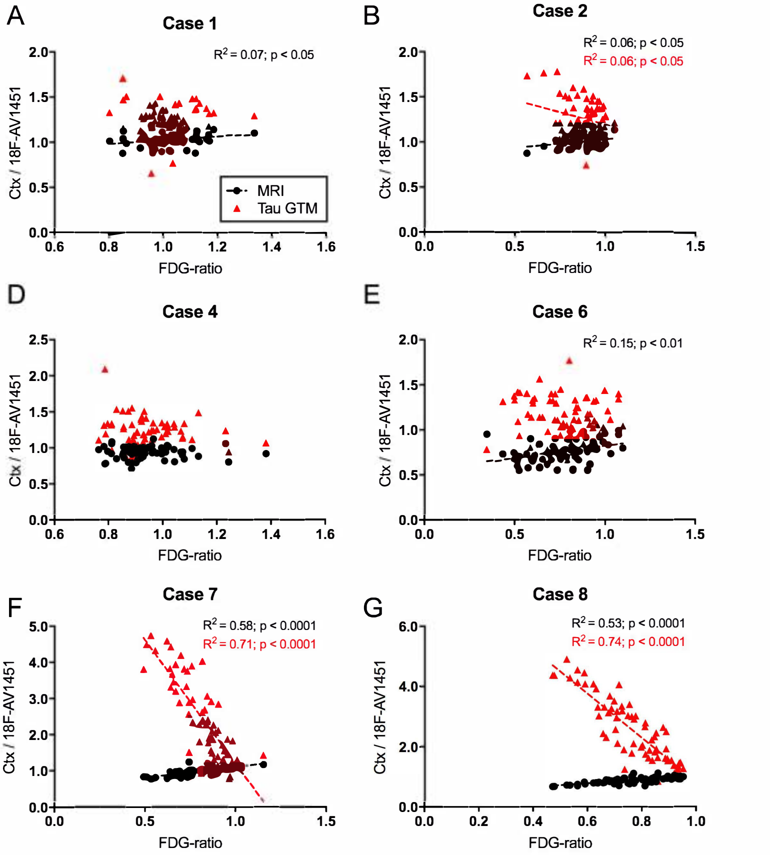

Supplemental Figure e-2.

Correlations of cortical thickness (black) and ^{18}F-AV-1451 retention (red) to ^{18}F-FDG ratio (Subject FDG/Control FDG) in four CBS-patients (cases 1, 2, 4 and 6) and two AD-subjects mimicking CBS (case 7 and 8). FDG was found to correlate to cortical thickness in all cases but case 4. FDG correlated inversely to ^{18}F-AV-1451 PET in the AD cases, but only weakly in CBS case 2.
