# Supplementary material for: In vivo retention of 18F-AV-1451 in corticobasal syndrome
Source: Neurology. 2017 Aug 22;89(8):845–53. doi: 10.1212/WNL.0000000000004264 (PMC5580862; doi:10.1212/WNL.0000000000004264)
Supplement: Data Supplement [file supp_WNL.0000000000004264_Suppl_Figure_e-3.pdf]

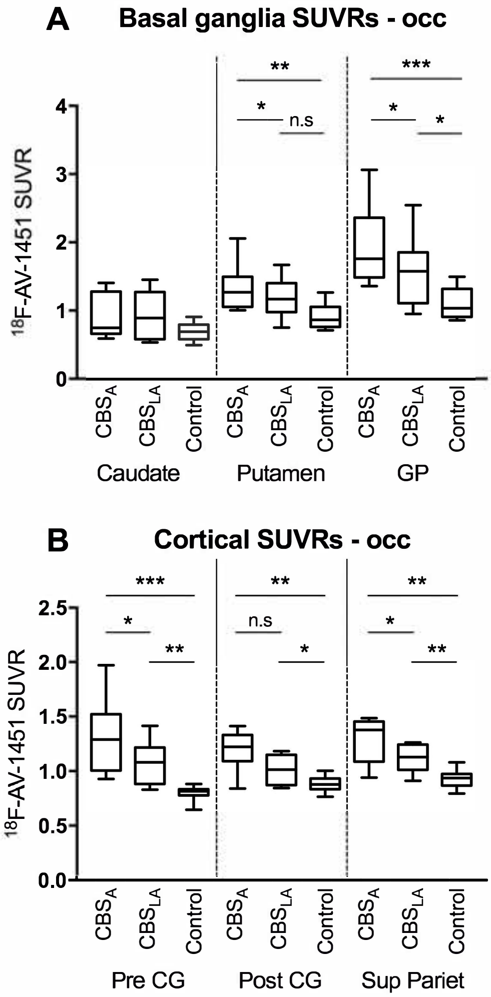

Supplementary Figure e-3

^{18}F-AV-1451 SUVRs in different brain regions with an occipital reference region. SUVRs are shown for the basal ganglia (A) and the cerebral cortex (B). Results are shown for CBS, most affected side (CBS_A); CBS less affected side (CBS_{LA}) and Controls. Pre CG - Precentral gyrus; Post CG - Postcentral gyrus; Sup Pariet - Superior Parietal gyrus. * p<0.05, ** p<0.01, *** p<0.001, n.s - not significant.
