# Supplementary material for: In vivo retention of 18F-AV-1451 in corticobasal syndrome
Source: Neurology. 2017 Aug 22;89(8):845–53. doi: 10.1212/WNL.0000000000004264 (PMC5580862; doi:10.1212/WNL.0000000000004264)
Supplement: Data Supplement [file supp_WNL.0000000000004264_Suppl_Figure_e-4.pdf]

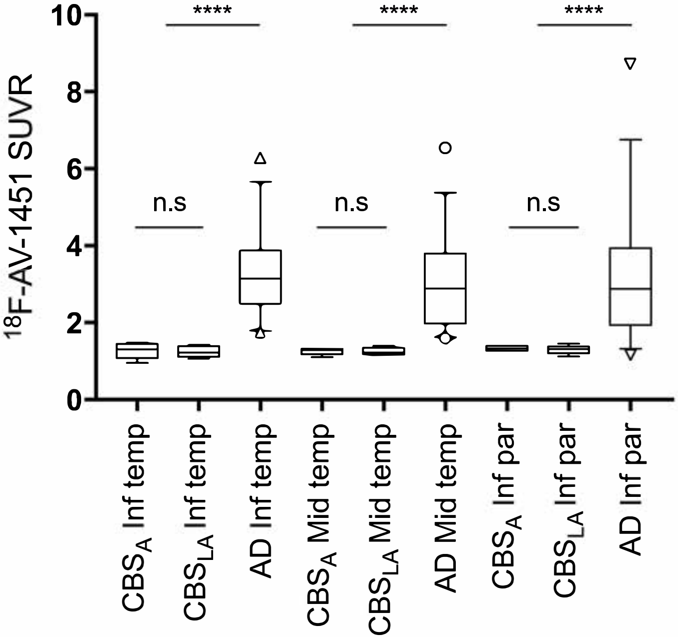

Supplemental Figure e-4.

Comparison of ^{18}F-SUVR retention in regions typically associated with AD pathology. A - affected side. Inf temp - inferior temporal gyrus. Inf par - inferior parietal gyrus. LA - less affected side. Mid temp - Middle temporal gyrus. ns - non significant. **** p < .0001.
