# Supplementary material for: In vivo retention of 18F-AV-1451 in corticobasal syndrome
Source: Neurology. 2017 Aug 22;89(8):845–53. doi: 10.1212/WNL.0000000000004264 (PMC5580862; doi:10.1212/WNL.0000000000004264)
Supplement: Data Supplement [file supp_WNL.0000000000004264_Supplementary_Table_e-2.pdf]

Supplementary Table e-2. Retention of 18F-AV-1451 in patients with CBS

	Affected side (n=6)	Less affected side (n=6)	Controls (n=17)
Precentral gyrus	1.09 ± 0.05	0.99 ± 0.04 *	0.91 ± 0.01 **
Postcentral gyrus	0.99 ± 0.04	0.90 ± 0.04 *	0.91 ± 0.02 *
Sup parietal gyrus	1.10 ± 0.05	1.01 ± 0.03 *	0.97 ± 0.02
Caudate	1.08 ± 0.07	1.08 ± 0.07	1.02 ± 0.04
Putamen	1.71 ± 0.08	1.55 ± 0.07 *	1.42 ± 0.05 **
Globus pallidus	2.12 ± 0.13	1.82 ± 0.11 *	1.59 ± 0.06 **
Most atrophic cortical region	1.22 ± 0.05	1.09 ± 0.03 *	0.91 ± 0.02§ ***

§ A control region from bilateral pre and post-central gyri was used for comparison in controls. Significance compared to CBS affected side, * p<0.05, ** p<0.01, *** p<0.001.
