# Supplementary material for: In vivo retention of 18F-AV-1451 in corticobasal syndrome
Source: Neurology. 2017 Aug 22;89(8):845–53. doi: 10.1212/WNL.0000000000004264 (PMC5580862; doi:10.1212/WNL.0000000000004264)
Supplement: Data Supplement [file supp_WNL.0000000000004264_e-Methods.docx]

**Supplement**

***In vivo* retention of ^18^F-AV-1451 in clinically diagnosed corticobasal degeneration**

Ruben Smith, Michael Schöll, Håkan Widner, Danielle van Westen, Per Svenningsson, Douglas Hägerström, Tomas Ohlsson, Jonas Jögi, Christer Nilsson, Oskar Hansson.

**Supplementary methods**

*PET analysis*

*Voxelwise analysis of PET and MRI data*

Voxel-based morphometry (VBM) as implemented in SPM12 (Wellcome Department of Cognitive Neurology, London, UK; [http://www.fil.ion.ucl.ac.uk/spm](https://email.ki.se/owa/redir.aspx?SURL=6njNG5OnL9TygKIsNXJ_bxA-T8dkViAacqXqhzRBUMRUxywLO1rTCGgAdAB0AHAAOgAvAC8AdwB3AHcALgBmAGkAbAAuAGkAbwBuAC4AdQBjAGwALgBhAGMALgB1AGsALwBzAHAAbQA.&URL=http%3a%2f%2fwww.fil.ion.ucl.ac.uk%2fspm)) was used to evaluate grey matter (GM) intensity differences as a measure of GM atrophy. In preparation, all individual T1-weighted MR images were segmented into tissue classes, and the GM segmentations subsequently warped into a common MNI152 standard space (using a cohort-specific template created with the DARTEL toolbox and Jacobian-scaling to estimate GM intensity). The resulting maps were smoothed with an 8 mm full-width at half maximum (FWHM) Gaussian kernel.

Using FSL (v5.0.6, http://fsl.fmrib.ox.ac.uk), we then created a mean and a standard deviation image based on the control group’s VBM and AV-1451 images that had been warped into a common MNI152 standard space employing their co-registered MRI scans and the above-mentioned cohort-specific template. Subsequently, we created a z-score map for the inverted VBM images (less GM intensity as a measure for GM atrophy) and the AV-1451 images for each CBD patient and mean z-score maps (AV-1451 and VBM) for the AD dementia patients.

Group differences in AV-1451 uptake were tested using the two-sample t-test implemented in SPM12 with participants’ age as covariate. Results were thresholded at a statistical significance level of p<0.05 corrected for family-wise error or p<0.001 uncorrected, respectively. The resulting t-maps were rendered and visualized on the FSL MNI152 brain template using Mango software (v4.0).

*Partial Volume Effects Correction*

Partial Volume Effects correction was performed using the Geometric Transfer Method (GTM) as previously described^1^, using FreeSurfer parcellations, smoothed with 5 mm FWHM to calculate transfers across ROI borders.

*Analysis of SUVRs in atrophic regions*

^18^F-AV-1451 PET-data for Figure 3C were generated using Pmod 3.703 (Pmod technologies Llc, Zürich, Switzerland). In brief, ^18^F-AV-1451 PET images and T1-MPRAGE images were coregistered using the NeuroTool and a cerebellar grey matter reference was generated using the Automatic Anatomical Labeling (AAL atlas). For assessing the ^18^F-AV-1451 retention in the atrophic cortex, ROIs were created in the areas judged visually to be the most atrophic using the T1-MPRAGE image by a person blinded to the PET data. A sample ROI (2,1 - 6, 5 cubic cm) in the atrophic region was created in the most affected side and a ROI of similar size was then created in a corresponding area in the contralateral hemisphere. The MRI images were then coregistered to the PET image and ^18^F-AV-1451 data extracted from these ROIs. A composite region of bilateral pre- and postcentral gyri was used for comparison in controls.

*Corticospinal tract analysis*

We applied the JHU ICBM tracts maxprob thr25 2mm DTI-based white matter atlas (as distributed with FSL v 5.0.9)^2, 3^ to the AV-1451 images in MNI space in order to extract regional AV-1451 SUVR in the bilateral cortical spinal tract.

*Segmented corticospinal tract analysis.*

The corticospinal tract was additionally segmented manually on transversal T1-mprage MRI images using pmod 3.703. In a normal control the subcortical white matter underlying the precentral gyrus as well as the posterior limb of the internal capsule from the appearance of the putamen and the thalamus on transversal images until the appearance of the anterior commissure were delineated bilaterally. These VOIs were then transformed to atlas space (normalized space). For each patient and control the VOIs in atlas space were then transformed back onto the subject MRI and the PET image coregistered into MRI space and SUVRs measured with a cerebellar grey matter reference.

*Occipital reference region*

As occipital reference in Suppl. Figure 1 Desikan-Killiany atlas regions cuneus, lingual, lateral occipital and pericalcarine cortex were pooled.

**References**

1. Rousset OG, Ma Y, Evans AC. Correction for partial volume effects in PET: principle and validation. J Nucl Med 1998;39:904-911.

2. Hua K, Zhang J, Wakana S, et al. Tract probability maps in stereotaxic spaces: analyses of white matter anatomy and tract-specific quantification. Neuroimage 2008;39:336-347.

3. Mori S, Crain BJ. MRI atlas of human white matter, 1st ed. Amsterdam ; Boston: Elsevier, 2005.
