# Supplementary material for: In vivo retention of 18F-AV-1451 in corticobasal syndrome
Source: Neurology. 2017 Aug 22;89(8):845–53. doi: 10.1212/WNL.0000000000004264 (PMC5580862; doi:10.1212/WNL.0000000000004264)
Supplement: Data Supplement [file supp_WNL.0000000000004264_Supplementary_Table_e-1.docx]

**Supplementary table e-1**

Clinical data of controls, AD dementia patients and PSP-patients

|  | **Age (years ± SD)** | **MMSE (Median; Range)** | **Number Aβ positive (%)** |
| --- | --- | --- | --- |
| Control (n=17) | 73±6 | 30 (27-30) | 5/17 |
| AD dementia (n=31) | 71±9 | 21 (7-27) | 31/31 |
| PSP (n= 11) | 71±7 | 29 (22-30) | N/A |

AD - Alzheimer's disease; MMSE - Mini-Mental State Exam; N/A - Not available; PSP - Progressive Supranuclear Palsy; SD - Standard deviation.
